# 8-Iodo-5,7-dimeth­oxy-4-methyl-2*H*-chromen-2-one

**DOI:** 10.1107/S1600536811007549

**Published:** 2011-03-05

**Authors:** P. S. Pereira Silva, Mehtab Parveen, Akhtar Ali, M. Ramos Silva

**Affiliations:** a CEMDRX, Physics Department, University of Coimbra, P-3004-516 Coimbra, Portugal; b Department of Chemistry, Aligarh Muslim University, Aligarh 202 002, India

## Abstract

In the title compound, C_12_H_11_IO_4_, the C and O atoms of both meth­oxy groups lie very close to the mean plane of the six C atoms of the benzene ring. The O and C atoms of the group lying closest to the I atom are 0.012 (3) and 0.022 (4) Å, respectively, out of the mean plane. For the other meth­oxy group, the corresponding distances are 0.020 (3) and 0.078 (4) Å. In the crystal, there are only very weak inter­molecular C—H⋯O hydrogen bonds and O⋯I contacts [3.080 (2) Å]. The mol­ecules are approximately parallel to (100), forming a layered structure.

## Related literature

For medicinal applications of coumarin derivatives, see: Lin *et al.* (2006 ▶); Massimo *et al.* (2003 ▶); Tyagi *et al.* (2003 ▶); Nawrot-Modranka *et al.* (2006 ▶); Sardari *et al.* (1999 ▶); Huang *et al.* (2005 ▶); Elinos-Baez *et al.* (2005 ▶). For the synthesis of the title compound, see: Ali & Ilyas (1986 ▶). For a similar structure, see: Pereira Silva *et al.* (2010 ▶).
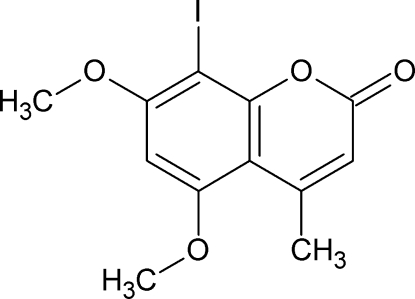


## Experimental

### 

#### Crystal data

C_12_H_11_IO_4_
*M* *_r_* = 346.11
Triclinic, 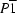

*a* = 7.1103 (7) Å
*b* = 9.5825 (10) Å
*c* = 9.9866 (9) Å
α = 109.645 (5)°
β = 94.734 (5)°
γ = 104.060 (5)°
*V* = 611.50 (10) Å^3^
*Z* = 2
Mo *K*α radiation
μ = 2.62 mm^−1^
*T* = 293 K
0.30 × 0.18 × 0.13 mm

#### Data collection

Bruker APEXII CCD area-detector diffractometer
Absorption correction: multi-scan (*SADABS*; Sheldrick, 2003 ▶) *T*_min_ = 0.511, *T*_max_ = 0.712
17329 measured reflections
2970 independent reflections
2701 reflections with *I* > 2σ(*I*)
*R*_int_ = 0.025

#### Refinement

*R*[*F* ^2^ > 2σ(*F* ^2^)] = 0.021
*wR*(*F* ^2^) = 0.090
*S* = 1.28
2970 reflections
157 parameters
H-atom parameters constrained
Δρ_max_ = 0.75 e Å^−3^
Δρ_min_ = −0.59 e Å^−3^

### 

Data collection: *APEX2* (Bruker, 2003 ▶); cell refinement: *SAINT* (Bruker, 2003 ▶); data reduction: *SAINT*; program(s) used to solve structure: *SHELXS97* (Sheldrick, 2008 ▶); program(s) used to refine structure: *SHELXL97* (Sheldrick, 2008 ▶); molecular graphics: *Mercury* (Macrae *et al.*, 2006 ▶); software used to prepare material for publication: *SHELXL97*.

## Supplementary Material

Crystal structure: contains datablocks global, I. DOI: 10.1107/S1600536811007549/fj2395sup1.cif

Structure factors: contains datablocks I. DOI: 10.1107/S1600536811007549/fj2395Isup2.hkl

Additional supplementary materials:  crystallographic information; 3D view; checkCIF report

## Figures and Tables

**Table 1:** Hydrogen-bond geometry (Å, °)

*D*—H⋯*A*	*D*—H	H⋯*A*	*D*⋯*A*	*D*—H⋯*A*
C6—H6⋯O2^i^	0.93	2.56	3.460 (4)	163
C13—H13*C*⋯O2^i^	0.96	2.51	3.211 (5)	130

Symmetry code: (i) 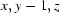.

**Fig. 1.**
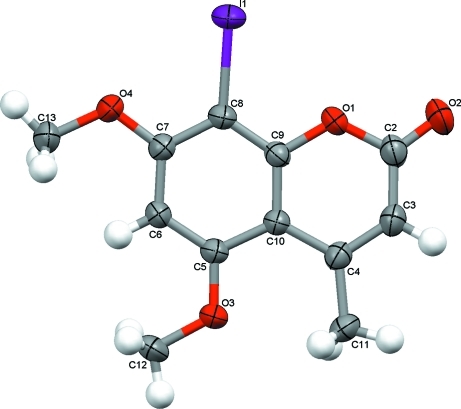
A plot of the title compound. Displacement ellipsoids are drawn at the 50% probability level.

**Fig. 2.**
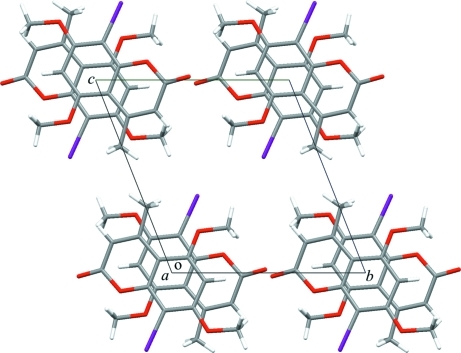
A packing diagram for (I), viewed down the a axis.

## supplementary crystallographic information

### Comment

Coumarin is the simplest member of the group of oxygen heterocyclics called benzo-2-pyrone. Coumarins are an important class of compounds due to their presence in natural products as well as their medicinal applications such as anti-inflammatory (Lin *et al.*, 2006), anti-viral (Massimo *et al.*, 2003), antioxidant (Tyagi *et al.*, 2003), antibacterial (Nawrot-Modranka *et al.*, 2006), antifungal (Sardari *et al.*, 1999), anti-HIV (Huang *et al.*, 2005) and as anti-carcinogenic (Elinos-Baez *et al.*, 2005). Besides the wide spectrum of biological applications of coumarin and its derivatives, there are also applications as cosmetics, optical brightening agents, and laser dyes. A recent report has revealed the anion sensing ability of some coumarin derivatives. Among various coumarin derivatives, recent pharmacological evaluation of iodocoumarins as cannabinoid receptor antagonists and inverse agonists has been done. Iodocoumarins such as 8-iodo-7-hydroxycoumarin exhibited moderate activity and 8-iodo-5,7-dihydroxycoumarin displayed good antimicrobial properties with MIC values <100 µg/ml. Also, iodocoumarins had been successfully used for the optimization of reaction conditions and kinetic studies in high throughput format. Because of the biological and pharmaceutical importance of iodocoumarins, several protocols for the synthesis have been reported.

In the light of the mentioned above we planned to synthesize iodocoumarins by reaction of 5,7-dimethoxy-4-methylcoumarin with iodine in basic media (Ali & Ilyas, 1986).

In the molecule of the title compound (Fig. 1), the best plane through the aromatic ring shows an r.m.s. deviation of 0.0059 Å; the O1—C2—C3—C4—C10—C9 ring shows a larger deviation from planarity, with an r.m.s. deviation of 0.0279 Å. The angle between these two planes is 2.85 (5)°.

The C2 atom of the carbonyl group has a distorted trigonal geometry with O2—C2—O1 [116.5 (3)°] and O2—C2—C3 [127.3 (23)°] deviating significantly from the ideal *sp*^2^ value of 120°.

The methoxy groups are almost in the same plane as the aromatic ring, as indicated by the torsion angles C12—O3—C5—C6 [1.8 (4)°] and C13—O4—C7—C6 [0.3 (4)°]. This contrasts with the geometry of 6,8-diiodo-5,7-dimethoxy-4-methylcoumarin (Pereira Silva *et al.*, 2010), where the methoxy groups are considerably out of this plane.

The iodine atom is approximately in the plane of the benzene ring and the methyl group is only slightly out of the pyrone ring plane.

In the crystal, the molecules are linked by very weak C—H···O hydrogen bonds and O···I contacts [3.080 (2) Å.]. There are no classic hydrogen bonds. The molecules are approximately parallel to (100), forming a layered structure (Fig. 2).

### Experimental

To a stirred solution of 5,7-dimethoxy-4-methylcoumarin (2.20 g, 10 mmol) in 15–20 ml of methanol containing 8.2 g KOH was dropwise added to a solution of I_2_ (2.56 g, 10 mmol) over a period of 30 min and stirred at room temperature for about 2 h. The reaction mixture was poured into water and residual iodine was removed by washing with sodium thiosulfate. On treatment with sodium thiosulfate we obtained a precipitate which was filtered and crystallized with CHCl_3_—MeOH as white crystals (300 mg, m.p. 490 K). This precipitate was identified as 6,8-Diiodo-5,7-dimethoxy-4-methylcoumarin (Pereira Silva *et al.*, 2010). The mother liquor showed the mixture of one major spot along with some minor impurity, which was removed by preparative thin layer chromatography (benzene:acetone; 3:2). The pure compound thus obtained was crystallized with CHCl_3_—MeOH as shining crystals of (I) (50 mg, m.p. 523 K).

### Refinement

All H atoms were located in a difference Fourier synthesis, placed in calculated positions and refined as riding on their parent atoms, using *SHELXL97* (Sheldrick, 2008) defaults.

### Crystal data

**Table:**

C_12_H_11_IO_4_	*Z* = 2
*M**_r_* = 346.11	*F*(000) = 336
Triclinic, *P*1	*D*_x_ = 1.880 Mg m^−^^3^
Hall symbol: -P 1	Melting point: 523 K
*a* = 7.1103 (7) Å	Mo *K*α radiation, λ = 0.71073 Å
*b* = 9.5825 (10) Å	Cell parameters from 5373 reflections
*c* = 9.9866 (9) Å	θ = 2.6–28.2°
α = 109.645 (5)°	µ = 2.62 mm^−^^1^
β = 94.734 (5)°	*T* = 293 K
γ = 104.060 (5)°	Irregular block, light pink
*V* = 611.50 (10) Å^3^	0.30 × 0.18 × 0.13 mm

### Data collection

**Table:**

Bruker APEXII CCD area-detector diffractometer	2970 independent reflections
Radiation source: fine-focus sealed tube	2701 reflections with *I* > 2σ(*I*)
graphite	*R*_int_ = 0.025
φ and ω scans	θ_max_ = 28.3°, θ_min_ = 2.2°
Absorption correction: multi-scan (*SADABS*; Sheldrick, 2003)	*h* = −9→9
*T*_min_ = 0.511, *T*_max_ = 0.712	*k* = −12→12
17329 measured reflections	*l* = −13→13

### Refinement

**Table:**

Refinement on *F*^2^	Primary atom site location: structure-invariant direct methods
Least-squares matrix: full	Secondary atom site location: difference Fourier map
*R*[*F*^2^ > 2σ(*F*^2^)] = 0.021	Hydrogen site location: inferred from neighbouring sites
*wR*(*F*^2^) = 0.090	H-atom parameters constrained
*S* = 1.28	*w* = 1/[σ^2^(*F*_o_^2^) + (0.0503*P*)^2^ + 0.1088*P*] where *P* = (*F*_o_^2^ + 2*F*_c_^2^)/3
2970 reflections	(Δ/σ)_max_ = 0.001
157 parameters	Δρ_max_ = 0.75 e Å^−^^3^
0 restraints	Δρ_min_ = −0.59 e Å^−^^3^

### Special details

**Table:**

Geometry. All e.s.d.'s (except the e.s.d. in the dihedral angle between two l.s. planes) are estimated using the full covariance matrix. The cell e.s.d.'s are taken into account individually in the estimation of e.s.d.'s in distances, angles and torsion angles; correlations between e.s.d.'s in cell parameters are only used when they are defined by crystal symmetry. An approximate (isotropic) treatment of cell e.s.d.'s is used for estimating e.s.d.'s involving l.s. planes.
Refinement. Refinement of *F*^2^ against ALL reflections. The weighted *R*-factor *wR* and goodness of fit *S* are based on *F*^2^, conventional *R*-factors *R* are based on *F*, with *F* set to zero for negative *F*^2^. The threshold expression of *F*^2^ > σ(*F*^2^) is used only for calculating *R*-factors(gt) *etc*. and is not relevant to the choice of reflections for refinement. *R*-factors based on *F*^2^ are statistically about twice as large as those based on *F*, and *R*- factors based on ALL data will be even larger.

### Fractional atomic coordinates and isotropic or equivalent isotropic displacement parameters (Å^2^)

**Table:**

	*x*	*y*	*z*	*U*_iso_*/*U*_eq_	
I1	0.85918 (3)	0.28855 (2)	0.398380 (19)	0.04982 (11)	
O1	0.7959 (3)	0.3031 (2)	0.0951 (2)	0.0420 (4)	
O2	0.7759 (5)	0.4881 (3)	0.0176 (3)	0.0674 (7)	
O3	0.6758 (4)	−0.2218 (2)	−0.2155 (2)	0.0476 (5)	
O4	0.7788 (4)	−0.0678 (3)	0.3021 (2)	0.0473 (5)	
C2	0.7757 (5)	0.3557 (4)	−0.0158 (4)	0.0463 (7)	
C3	0.7564 (5)	0.2457 (4)	−0.1588 (3)	0.0448 (6)	
H3	0.7581	0.2808	−0.2350	0.054*	
C4	0.7360 (4)	0.0940 (3)	−0.1890 (3)	0.0378 (5)	
C5	0.7146 (4)	−0.1155 (3)	−0.0803 (3)	0.0345 (5)	
C6	0.7277 (4)	−0.1539 (3)	0.0418 (3)	0.0357 (5)	
H6	0.7109	−0.2565	0.0317	0.043*	
C7	0.7659 (4)	−0.0396 (3)	0.1791 (3)	0.0359 (5)	
C8	0.7918 (4)	0.1150 (3)	0.1944 (3)	0.0356 (5)	
C9	0.7759 (4)	0.1501 (3)	0.0716 (3)	0.0335 (5)	
C10	0.7408 (4)	0.0401 (3)	−0.0695 (3)	0.0328 (5)	
C11	0.7118 (5)	−0.0087 (4)	−0.3441 (3)	0.0468 (7)	
H11A	0.7269	0.0532	−0.4030	0.070*	
H11B	0.5830	−0.0819	−0.3742	0.070*	
H11C	0.8100	−0.0630	−0.3545	0.070*	
C12	0.6410 (6)	−0.3812 (4)	−0.2349 (4)	0.0552 (8)	
H12A	0.7577	−0.3960	−0.1932	0.083*	
H12B	0.6080	−0.4433	−0.3362	0.083*	
H12C	0.5340	−0.4114	−0.1882	0.083*	
C13	0.7526 (6)	−0.2253 (4)	0.2918 (4)	0.0543 (8)	
H13A	0.6194	−0.2857	0.2470	0.081*	
H13B	0.7790	−0.2272	0.3869	0.081*	
H13C	0.8418	−0.2677	0.2348	0.081*	

### Atomic displacement parameters (Å^2^)

**Table:**

	*U*^11^	*U*^22^	*U*^33^	*U*^12^	*U*^13^	*U*^23^
I1	0.06807 (17)	0.03768 (14)	0.03117 (13)	0.01100 (10)	0.00271 (9)	0.00155 (9)
O1	0.0595 (12)	0.0256 (9)	0.0397 (11)	0.0125 (8)	0.0081 (9)	0.0105 (8)
O2	0.110 (2)	0.0386 (13)	0.0677 (17)	0.0334 (14)	0.0261 (16)	0.0265 (13)
O3	0.0788 (15)	0.0293 (10)	0.0298 (10)	0.0149 (10)	0.0061 (9)	0.0062 (8)
O4	0.0766 (14)	0.0390 (11)	0.0294 (10)	0.0203 (10)	0.0069 (9)	0.0144 (9)
C2	0.0567 (16)	0.0366 (15)	0.0523 (18)	0.0160 (12)	0.0147 (14)	0.0217 (14)
C3	0.0577 (16)	0.0417 (16)	0.0433 (16)	0.0159 (13)	0.0127 (13)	0.0239 (13)
C4	0.0415 (13)	0.0402 (14)	0.0350 (14)	0.0121 (11)	0.0082 (10)	0.0175 (12)
C5	0.0409 (12)	0.0300 (12)	0.0297 (13)	0.0109 (10)	0.0054 (10)	0.0073 (10)
C6	0.0454 (13)	0.0277 (12)	0.0340 (13)	0.0114 (10)	0.0070 (10)	0.0112 (10)
C7	0.0422 (13)	0.0351 (13)	0.0314 (13)	0.0126 (10)	0.0052 (10)	0.0127 (11)
C8	0.0429 (13)	0.0300 (12)	0.0296 (13)	0.0100 (10)	0.0043 (10)	0.0066 (10)
C9	0.0366 (12)	0.0266 (12)	0.0346 (13)	0.0081 (9)	0.0057 (10)	0.0086 (10)
C10	0.0385 (12)	0.0294 (12)	0.0295 (12)	0.0097 (10)	0.0059 (9)	0.0099 (10)
C11	0.0625 (17)	0.0474 (17)	0.0293 (14)	0.0130 (14)	0.0084 (12)	0.0149 (13)
C12	0.089 (2)	0.0292 (14)	0.0413 (17)	0.0170 (15)	0.0137 (16)	0.0049 (13)
C13	0.087 (2)	0.0440 (17)	0.0436 (17)	0.0286 (17)	0.0147 (16)	0.0233 (15)

### Geometric parameters (Å, °)

**Table:**

I1—C8	2.078 (3)	C6—C7	1.392 (4)
O1—C2	1.374 (4)	C6—H6	0.9300
O1—C9	1.374 (3)	C7—C8	1.401 (4)
O2—C2	1.198 (4)	C8—C9	1.380 (4)
O3—C5	1.346 (3)	C9—C10	1.405 (4)
O3—C12	1.429 (4)	C11—H11A	0.9600
O4—C7	1.345 (3)	C11—H11B	0.9600
O4—C13	1.441 (4)	C11—H11C	0.9600
C2—C3	1.433 (5)	C12—H12A	0.9600
C3—C4	1.350 (4)	C12—H12B	0.9600
C3—H3	0.9300	C12—H12C	0.9600
C4—C10	1.452 (4)	C13—H13A	0.9600
C4—C11	1.498 (4)	C13—H13B	0.9600
C5—C6	1.389 (4)	C13—H13C	0.9600
C5—C10	1.423 (4)		
			
C2—O1—C9	122.5 (2)	O1—C9—C8	115.4 (2)
C5—O3—C12	118.9 (2)	O1—C9—C10	120.7 (2)
C7—O4—C13	118.3 (2)	C8—C9—C10	123.9 (2)
O2—C2—O1	116.5 (3)	C9—C10—C5	115.6 (2)
O2—C2—C3	127.3 (3)	C9—C10—C4	118.2 (2)
O1—C2—C3	116.3 (2)	C5—C10—C4	126.3 (2)
C4—C3—C2	123.6 (3)	C4—C11—H11A	109.5
C4—C3—H3	118.2	C4—C11—H11B	109.5
C2—C3—H3	118.2	H11A—C11—H11B	109.5
C3—C4—C10	118.2 (3)	C4—C11—H11C	109.5
C3—C4—C11	117.9 (3)	H11A—C11—H11C	109.5
C10—C4—C11	123.9 (3)	H11B—C11—H11C	109.5
O3—C5—C6	122.7 (2)	O3—C12—H12A	109.5
O3—C5—C10	115.8 (2)	O3—C12—H12B	109.5
C6—C5—C10	121.5 (2)	H12A—C12—H12B	109.5
C5—C6—C7	120.3 (2)	O3—C12—H12C	109.5
C5—C6—H6	119.8	H12A—C12—H12C	109.5
C7—C6—H6	119.8	H12B—C12—H12C	109.5
O4—C7—C6	123.8 (2)	O4—C13—H13A	109.5
O4—C7—C8	116.3 (2)	O4—C13—H13B	109.5
C6—C7—C8	119.9 (2)	H13A—C13—H13B	109.5
C9—C8—C7	118.7 (2)	O4—C13—H13C	109.5
C9—C8—I1	120.70 (19)	H13A—C13—H13C	109.5
C7—C8—I1	120.6 (2)	H13B—C13—H13C	109.5
			
C9—O1—C2—O2	172.5 (3)	C2—O1—C9—C8	−177.2 (3)
C9—O1—C2—C3	−7.4 (4)	C2—O1—C9—C10	2.5 (4)
O2—C2—C3—C4	−172.5 (4)	C7—C8—C9—O1	177.7 (2)
O1—C2—C3—C4	7.4 (5)	I1—C8—C9—O1	−3.7 (3)
C2—C3—C4—C10	−2.2 (4)	C7—C8—C9—C10	−1.9 (4)
C2—C3—C4—C11	178.4 (3)	I1—C8—C9—C10	176.7 (2)
C12—O3—C5—C6	1.8 (4)	O1—C9—C10—C5	−177.5 (2)
C12—O3—C5—C10	−177.9 (3)	C8—C9—C10—C5	2.1 (4)
O3—C5—C6—C7	−179.2 (3)	O1—C9—C10—C4	3.0 (4)
C10—C5—C6—C7	0.4 (4)	C8—C9—C10—C4	−177.3 (2)
C13—O4—C7—C6	0.3 (4)	O3—C5—C10—C9	178.3 (2)
C13—O4—C7—C8	179.8 (3)	C6—C5—C10—C9	−1.3 (4)
C5—C6—C7—O4	179.3 (3)	O3—C5—C10—C4	−2.2 (4)
C5—C6—C7—C8	−0.2 (4)	C6—C5—C10—C4	178.1 (3)
O4—C7—C8—C9	−178.6 (2)	C3—C4—C10—C9	−3.1 (4)
C6—C7—C8—C9	0.9 (4)	C11—C4—C10—C9	176.3 (3)
O4—C7—C8—I1	2.8 (3)	C3—C4—C10—C5	177.5 (3)
C6—C7—C8—I1	−177.7 (2)	C11—C4—C10—C5	−3.1 (4)

### Hydrogen-bond geometry (Å, °)

**Table:**

*D*—H···*A*	*D*—H	H···*A*	*D*···*A*	*D*—H···*A*
C6—H6···O2^i^	0.93	2.56	3.460 (4)	163
C13—H13C···O2^i^	0.96	2.51	3.211 (5)	130

Symmetry codes: (i) *x*, *y*−1, *z*.

## References

[bb1] Ali, S. M. & Ilyas, M. (1986). *J. Org. Chem.* **51**, 5415–5417.

[bb2] Bruker (2003). *APEX2* and *SAINT* Bruker AXS Inc., Madison, Wisconsin, USA.

[bb3] Elinos-Baez, C. M., Leon, F. & Santos, E. (2005). *Cell Biol. Int.* **29**, 703–708. 10.1016/j.cellbi.2005.04.003 15964220

[bb4] Huang, L., Yuon, X., Yu, D., Lee, K. H. & Chin, H. C. (2005). *Virology*, **332**, 623–628. 10.1016/j.virol.2004.11.033 15680427

[bb5] Lin, C. M., Huang, S. T., Lee, F. W., Sawkuo, H. & Lin, M. H. (2006). *Bioorg. Med. Chem.* **14**, 4402–4409. 10.1016/j.bmc.2006.02.042 16540334

[bb6] Macrae, C. F., Edgington, P. R., McCabe, P., Pidcock, E., Shields, G. P., Taylor, R., Towler, M. & van de Streek, J. (2006). *J. Appl. Cryst.* **39**, 453–457.

[bb7] Massimo, C., Francesco, E., Federica, M., Carla, M. M., Prieto, G. S. & Carlos, R. J. (2003). *Aust. J. Chem.* **56**, 59–60.

[bb8] Nawrot-Modranka, J., Nawrot, E. & Graczyk, J. (2006). *Eur. J. Med. Chem.* **41**, 1301–1309. 10.1016/j.ejmech.2006.06.004 16904795

[bb9] Pereira Silva, P. S., Parveen, M., Khanam, Z., Ali, A. & Ramos Silva, M. (2010). *Acta Cryst.* E**66**, o988. 10.1107/S1600536810011360 PMC2983982 21580784

[bb10] Sardari, S., Mori, Y., Horita, K., Micetich, R. G., Nishibe, S. & Daneshtalab, M. (1999). *Bioorg. Med. Chem.* **7**, 1933–1940. 10.1016/s0968-0896(99)00138-8 10530942

[bb11] Sheldrick, G. M. (2003). *SADABS* University of Göttingen, Germany.

[bb12] Sheldrick, G. M. (2008). *Acta Cryst.* A**64**, 112–122. 10.1107/S0108767307043930 18156677

[bb13] Tyagi, A. K., Raj, H. G., Vohra, P., Gupta, G., Kumari, R., Kumar, P. & Gupta, R. K. (2003). *Eur. J. Med. Chem.* **40**, 413–420. 10.1016/j.ejmech.2004.09.002 15804541

